# *Helicobacter pylori* (*H. pylori*) risk factor analysis and prevalence prediction: a machine learning-based approach

**DOI:** 10.1186/s12879-022-07625-7

**Published:** 2022-07-28

**Authors:** Van Tran, Tazmilur Saad, Mehret Tesfaye, Sosina Walelign, Moges Wordofa, Dessie Abera, Kassu Desta, Aster Tsegaye, Ahmet Ay, Bineyam Taye

**Affiliations:** 1 Department of Mathematics, Colgate University, 13 Oak Dr., Hamilton, NY USA; 2 College of Health Sciences, Department of Medical Laboratory Science, Addis Ababa University, Addis Ababa, Ethiopia; 3 Department of Biology, Colgate University, 13 Oak Dr., Hamilton, NY USA

**Keywords:** Machine learning, *H. pylori* infection, Classification, Feature selection, Logistic regression, School children, Ethiopia

## Abstract

**Background:**

Although previous epidemiological studies have examined the potential risk factors that increase the likelihood of acquiring *Helicobacter pylori* infections, most of these analyses have utilized conventional statistical models, including logistic regression, and have not benefited from advanced machine learning techniques.

**Objective:**

We examined *H. pylori* infection risk factors among school children using machine learning algorithms to identify important risk factors as well as to determine whether machine learning can be used to predict *H. pylori* infection status.

**Methods:**

We applied feature selection and classification algorithms to data from a school-based cross-sectional survey in Ethiopia. The data set included 954 school children with 27 sociodemographic and lifestyle variables. We conducted five runs of tenfold cross-validation on the data. We combined the results of these runs for each combination of feature selection (e.g., Information Gain) and classification (e.g., Support Vector Machines) algorithms.

**Results:**

The XGBoost classifier had the highest accuracy in predicting *H. pylori* infection status with an accuracy of 77%—a 13% improvement from the baseline accuracy of guessing the most frequent class (64% of the samples were *H. Pylori* negative.) K-Nearest Neighbors showed the worst performance across all classifiers. A similar performance was observed using the F1-score and area under the receiver operating curve (AUROC) classifier evaluation metrics. Among all features, place of residence (with urban residence increasing risk) was the most common risk factor for *H. pylori* infection, regardless of the feature selection method choice. Additionally, our machine learning algorithms identified other important risk factors for *H. pylori* infection, such as; electricity usage in the home, toilet type, and waste disposal location. Using a 75% cutoff for robustness, machine learning identified five of the eight significant features found by traditional multivariate logistic regression. However, when a lower robustness threshold is used, machine learning approaches identified more *H. pylori* risk factors than multivariate logistic regression and suggested risk factors not detected by logistic regression.

**Conclusion:**

This study provides evidence that machine learning approaches are positioned to uncover *H. pylori* infection risk factors and predict *H. pylori* infection status. These approaches identify similar risk factors and predict infection with comparable accuracy to logistic regression, thus they could be used as an alternative method.

**Supplementary Information:**

The online version contains supplementary material available at 10.1186/s12879-022-07625-7.

## Introduction

*Helicobacter pylori* is a gram-negative bacterium that resides in the stomach and can cause inflammation leading to long-term effects, such as gastric ulcers, cancer, and lymphoma of the stomach mucosal linings [1–3]. The approximate global prevalence of *H. pylori* infection is 50%, but infection rates vary between developed and developing countries, often ranging between 20–80%, with developing countries having higher infection rates [1–5]. Previous research and analysis of *H. pylori* prevalence in populations have examined common risk factors that increase the likelihood of acquiring the bacteria. Some of the most commonly identified risk factors across various studies in different geographic populations are larger family size, less education, lower socioeconomic status, less frequent hygiene practices, and lower sanitation with specific emphasis on sources of water and defecation [4, 6–10]. The extent to which common risk factors influence the acquisition of *H. pylori* can depend on cultural and geographical circumstances, giving rise to the large variation in prevalence globally [3, 11, 12]. Although, majority of *H*. *pylori* infected individuals do not show clinical symptoms[13], untreated infections could last a lifetime leading to chronic gastritis, which can develop into ulcer and carcinoma[14]. This necessitates the need for early diagnosis and proper disease therapy. Understanding the occurrence of H. pylori infection and its interaction with socio-demographic factors is important in developing an effective *H.* *pylori* infection risk management tools tailored to local public health policy.

Our group's recent study also identified a set of sociodemographic risk factors associated with *H. pylori* infection among children in Ethiopia [15]. These studies have utilized conventional statistical models such as logistic regression to identify risk factors and have not benefitted from advanced techniques from machine learning. While logistic regression is a rudimentary form of machine learning, it is not designed to examine large, highly correlated sets of predictors or to elucidate interactions among predictors without a priori specification [16]. Additional research using flexible modeling procedures that capture the complexities of the *H. pylori* risk factors is needed.

To our knowledge, no study has attempted to investigate *H. pylori* risk factors using an advanced machine learning approach. Machine learning is a branch of artificial intelligence based on the idea that computers can learn from data, identify patterns, and make decisions with minimal human intervention [17]. Compared with an epidemiological and statistical approach that requires strong data assumptions, machine learning can examine data to identify an underlying structure using an iterative process to learn from the data [16] and identify potential risk factors associated with a disease without any user input bias. Thus, using a diverse set of advanced machine learning algorithms can improve the confidence in the findings from traditional methods.

Moreover, they can also be used for their predictive use. In this study, we used data from a detailed Ethiopian school children survey to assess risk factors for *H. pylori* infection and develop predictive disease prevalence models using advanced machine learning approaches, and compared it with a traditional statistical approach (logistic regression).

## Methods

### Data source and sample selection

**Fig. 1:**
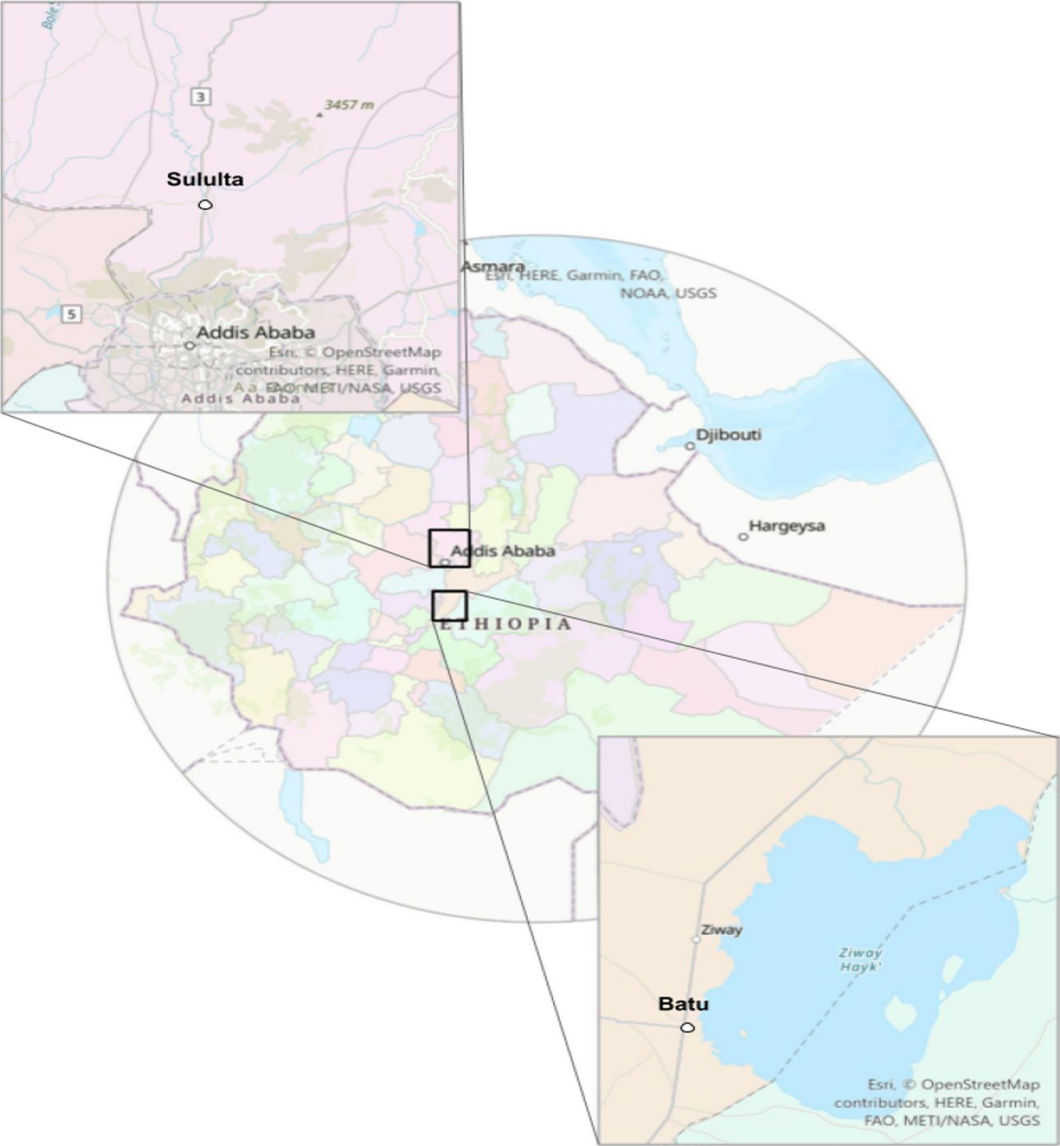
Map of Sululta and Ziway (Batu) can be located to the north and south of Addis Ababa, respectively, Ethiopia

We conducted a two-part cross-sectional survey among school children in the Oromia region of Ethiopia in the towns of Ziway and Sululta in 2016 and 2017 (Fig. 1). This area extends north and south by 30 km and 160 km, respectively, from the capital city of Addis Ababa. Five elementary schools, three (i.e., Laga Dima, Wasarbi, and Abdi Boru) from Sululta and two schools (i.e., Sher and Batu) from Ziway town were included in the study. Following written consent documentation by parents or legal guardians of the children, we collected demographic data concerning lifestyle and behavior from a total of 954 study subjects using an interview-led questionnaire.

### *H. pylori* detection

A more detailed description of H pylori measurements can be found in our previous published studies [18, 19]. Briefly, each participant provided a stool and blood sample for H. pylori testing. A widely used non-invasive rapid Helicobacter pylori (Cassette) stool antigen test (Diagnostic Automation Inc., USA) was used to detect *H. pylori* antigen in stool samples as indicative of recent infection. Additionally, a rapid antibody test was used to detect past or current infection in serum samples. *H pylori* positivity was defined by a positive result for either *H. pylori *antigen or antibody test for this study.

### Machine learning algorithms

#### Data preprocessing

We excluded any risk factor we missed more than 5% of the lifestyle and behavior data. In addition, we normalized data for risk factors that had continuous values and transformed factors with multiple categories using one-hot encoding. For the latter, one category for each factor was dropped to avoid multicollinearity. The preprocessed data included 954 samples (91 children aged 0–5, 425 aged 6–10, and 438 aged 11–15) with 27 risk factors of *H. pylori* (Table 1).

**Table 1:** List of risk factors (features) used in the study and the survey results

Feature	Response	*H. pylori* ( +)	*H. pylori* (−)
Residence	**Urban**	293 (54.3%)	247 (45.7%)
	Rural	50 (12.1%)	364 (87.9%)
Allergies	Any allergic disease	70 (31%)	156 (69%)
	**No allergies**	273 (37.5%)	455 (62.5%)
Parasites	Any parasites found	110 (37.2%)	186 (62.8%)
	**No parasites**	233 (35.4%)	425 (64.6%)
Cooking area	**Inside house**	276 (33.5%)	547 (66.5%)
	Outside house	67 (51.1%)	64 (48.9%)
Dewormed status	**Dewormed**	247 (33.7%)	487 (66.3%)
	Not dewormed	96 (43.6%)	124 (56.4%)
Cow	Family owns cow(s)	47 (23.4%)	154 (76.6%)
	**No cow(s)**	296 (39.3%)	457 (60.7%)
Smoking	**Smoker in household**	10 (16.4%)	51 (83.6%)
	No smokers	333 (37.3%)	560 (62.7%)
Cat	**No cat**	257 (38.6%)	408 (61.4%)
	Cat lives inside	53 (25.4%)	156 (74.6%)
	Cat lives outside	33 (41.3%)	47 (58.7%)
Dog	**No dog**	228 (40.1%)	341 (59.9%)
	Dog lives inside	0 (0%)	4 (100%)
	Dog lives outside	115 (30.2%)	266 (69.8%)
Electricity use	**Every day**	273 (55.2%)	222 (44.8%)
	Sometimes	11 (12.8%)	75 (87.2%)
	Never	59 (15.8%)	314 (84.2%)
Floor in Home	**Cement**	150 (36.8%)	258 (63.2%)
	Wood	4 (20%)	16 (80%)
	Mud	186 (35.9%)	332 (64.1%)
	Other	3 (37.5%)	5(62.5%)
Waste disposal	**Garbage bin**	80 (44.2%)	101 (55.8%)
	Pit	56 (36.6%)	97 (63.4%)
	Open field	26 (12.6%)	181 (87.4%)
	Burn	181 (43.8%)	232 (56.2%)
Age	**0–5 years**	43 (47.3%)	48 (52.7%)
	6–10 years	167 (39.3%)	258 (60.7%)
	11–15 years	133 (30.4%)	305 (69.6%)
Family size	**0–3 people**	55 (34.6%)	104 (65.4%)
	4–5 people	165 (35.6%)	299 (64.4%)
	> 5 people	123 (37.2%)	208 (62.8%)
Toilet	**Flush toilet**	10 (25%)	30 (75%)
	Pit toilet	325 (38.7%)	514 (61.3%)
	Open field	8 (10.7%)	67 (89.3%)
Water source	**Piped**	327 (38.3%)	526 (61.7%)
	Well	12 (15.4%)	66 (84.6%)
	River or rain water	4 (17.4%)	19 (82.6%)

The reference group is the one that is bolded in the response column

#### Feature selection

We used feature selection to reduce the number of redundant features (*H. pylori* risk factors in our study) and, subsequently, our dataset's dimensionality to detect the most important *H. pylori* risk factors and to improve the accuracy of our classifiers; machine learning algorithms that utilize data to understand how given risk factors relate to disease status.

First, we used ranking-based methods Information Gain (IG) and ReliefF (ReF) to assess each feature's importance independent of the other features using a scoring metric [20, 21]. Information gain (IG) uses mutual information between the feature and the target variable to calculate their mutual dependence. ReliefF (ReF) uses the Manhattan distance to calculate each feature's weight by randomly selecting samples from the dataset and estimating the distance between the nearby samples and their classes (*H. pylori* infection status in our study). Then, we ranked features based on these scores and chose the top 10 or 20 features for our classification runs. Since these ranking-based methods do not inherently have any way of discerning the interaction and confounding effects between risk factors, we have decided to remove the features beyond the 20^th^ risk factor.

Next, we used the subset-based methods Correlation-based Feature Selection (CFS), Minimum Redundancy Maximum Relevance (MRMR), Fast Correlation Based Filter (FCBF), and Sequential Forward Selection (SFS) [22–25] to find the optimal subset of features to predict *H. pylori* status. CFS, MRMR, and FCBF aim to find subsets of the feature space highly correlated to the target variable but uncorrelated amongst themselves based on different metrics in order to minimize the interaction effects between selected features. We used symmetrical uncertainty to evaluate CFS and FCBF. Our MRMR runs used basic scoring criteria of a linear combination of Shannon information terms. Finally, we specifically used a variant of SFS called Sequential Floating Forward Selection (SFFS). SFFS is a wrapper-based method that reduces the feature space by adding features one by one, calculating the classifier's performance, and using it as a metric to find the optimum feature subset.

#### Classifiers & hypertuning

We used the optimum feature subset to train a wide assortment of classifiers on the dataset to find which classifier maximized the accuracy of our *H. pylori* status predictions and to ensure they were robust across various feature selection methods. Specifically, we tested: K-Nearest Neighbors (KNN), Logistic Regression with Lasso penalty (LR), Support Vector Machines (SVM), Random Forests (RF), Naive Bayes (NB), and XGBoost (XGB) [26–31]. Bagging and boosting are ensemble methods that either combine multiple weak estimators or bootstrap the dataset and aggregate the results [32, 33]. We used boosting (with AdaBoost) and bagging to improve the prediction performance of our classifiers. The majority of the classifiers that we have used either inherently penalize confounding factors via regularization or are effective at handling interactive effects.

We used accuracy, F1-score, and area under the receiver operating characteristic curve (AUROC) as performance metrics since they provide a complementary view of classifiers' performance. Accuracy score is a better metric if true positives and true negatives are more important than false positives and false negatives. In contrast, F1-score is better when false positives and false negatives are more crucial. Also, F1-score and AUROC are better metrics than accuracy when there is an imbalanced class distribution (*H. pylori* status in our study). Our data showed a slight imbalance in the class distributions (64% of the samples were *H. pylori* negative). The baseline accuracy was calculated using the majority class prevalence, which refers to *H. pylori* negative cases. In this case, our baseline accuracy was 64%. Theoretically, the imbalance may cause the feature selection methods and classifiers to be biased towards the majority class. We repeated our study on our dataset up-sampled with the Synthetic Minority Oversampling (SMOTE) technique which uses the k-nearest neighbors algorithm to generate new samples and ensure that the dataset is balanced [34].

Each classifier uses different criteria and methods to fit itself onto the dataset—for instance, KNN classifies each sample based on the distribution of its neighbors, while XGBoost uses gradient boosted decision trees to do the same—but the exact details of algorithms and their implementations are beyond the scope of this paper [35, 36]. Each classifier also contains various parameters that need to be tuned based on the dataset to improve its performance. We first found suitable hyperparameter ranges for each classifier by running preliminary tests. Then we conducted a grid search on those hyperparameters to find the best combination. We did not run hyperparameter tuning on bagging and boosting ensemble methods due to the infeasible computational complexity. Thus, the ensemble classification was performed using the parameter combination for each estimator that gave the best classification results. For similar reasons, we did not run boosting with the SFFS feature selection method. Finally, boosting is part of the XGBoost classifier; thus, we did not conduct boosting with XGBoost.

#### Model validation

We then used tenfold cross-validation to determine the generalizability of the classifiers into other independent datasets. First, we split the dataset into ten different stratified folds, maintaining the distribution of the *H. pylori* classes across each fold. We imputed the missing data for each fold using an iterative imputer, which treats each feature being imputed as a dependent variable and fits a regression model based on the other features to predict the missing values [37]. Next, we selected the most important features using feature selection methods discussed above, then used data for those features to train the six classifiers for each hyperparameter combination. Since SFFS depends on the classifier and the fold itself, for the SFFS feature selection method, we ran feature selection with each classifier and hyperparameter combination. To make sure our results were robust, we repeated this tenfold cross-validation process five times. We used a specific seed for each repeat to ensure that the splitting of the dataset is the same for the SFFS and ensemble runs as well. In this way, we ensured that our results did not vary significantly by the way we split the data. We reported the highest accuracy and F1 score from the five runs with the corresponding classifier hyperparameter combination and feature selection method. We also calculated the AUROC score for each model using the best hyperparameter combination for each classifier. However, since we did not test our model on an independent dataset it could theoretically be overfit. Thus, we then repeated our study using nested cross validation. We first split the dataset into five subsets. In each iteration, one subset is used as test data and the other four are used for training. As mentioned earlier in this section, we then perform tenfold cross validation on the training data to select the best combination of hyperparameters for each classifier which is then used for evaluation on the unseen test data. However, nested cross validation is extremely computationally expensive and thus we were not able to run it with the SFFS feature selection method.

#### Clustering

We used two-dimensional hierarchical clustering to group the classifier and feature selection methods based on accuracy and F1 score [38]. We also used the same approach to group features and feature selection methods based on the frequency of each feature being selected in five ten-fold cross-validation runs. We presented the hierarchical clustering results as heatmaps.

#### Logistic regression model

We also created a logistic regression model (without Lasso) to compare our machine learning-based methods to the conventional model used throughout epidemiology. We preprocessed the dataset similar to the machine learning approach, removing unusable features, imputing values, and splitting nominal variables into different categories. We then ran two separate regression models: a stepwise multivariate logistic regression model using the Akaike information criterion and a univariate logistic regression model with each predictor. We then recorded the p-values and the odds-ratios for each of the regressions.

#### Code availability

The study's code was written in Python and R due to their ease of use and advanced statistical learning libraries. We will make our source code available upon request.

## Results

### Study population characteristics

In the sample of 954 children for which there were blood and stool sample data, 55.3% (526) were female, and 56.6% (540) lived in urban areas. There were 91 children aged 0–5, 425 aged 6–10, and 438 aged 11–15. The most common family size was 4–5 people (48.5%). A majority of families had some access to electricity (60.8%) but few used gas (10.9%). A majority of the children (76.9%, 732) had been dewormed in the past six months, 30.7% (293) of the children had parasites detected in their samples, and 35.9% (343) tested positive (current and past) *H. pylori* infection. See Table 1 for the full survey results.

### Logistic regression

Stepwise logistic regression models showed a significant increase in the odds of *H. pylori* infections among children who had no history of deworming treatment (feature 5; AOR = 1.68, 95% CI: 1.16–2.42, p = 0.006), who had smokers in the house (feature 7; AOR = 2.83, 95% CI:1.35–6.42, p = 0.008), who had pit latrine (feature 24; AOR = 2.34, 95% CI: 1.34–4.24, p = 0.004). A similar trend of increasing odds ratio was observed between infections with *H. pylori* and having a family size greater than 5 (feature 23), but this did not achieve statistical significance in the multivariate analyses (AOR = 1.30, 95% CI: 0.93–1.82, p = 0.12). In contrast, families who reported dumping their waste in an open field (feature 18), having a cow (feature 6), living in a rural area (feature 1), and sometimes or never using electricity (features 12–13) were inversely associated with *H. pylori* infection (Table 2). Some features had an odds ratio of greater than 1 in the univariate analysis but not retained the multivariate analysis.

**Table 2:** Univariate and stepwise multivariate logistic regression model of H. pylori risk factors in association with *H. pylori* infection in school children, Ethiopia

Feature	Meaning	*H. Pylori* ( +)	*H. Pylori* (−)	p-value (Uni)	COR	CI 95%	p-value (multi)	AOR	CI 95%
Residence	Place of residence								
0	Urban	293 (54.3%)	247 (45.7%)						
1	Rural	50 (12.1%)	364 (87.9%)	0.000	0.117	0.083–0.164	0.000	0.216	0.144–0.321
Allergy	Any allergic disease								
0	No allergy	273 (37.5%)	455 (62.5%)						
1	Allergy	70 (31%)	156 (69%)	0.075	0.748	0.543–1.029			
Parasite found	Any parasites found								
0	No allergy	233 (35.4%)	425 (64.6%)						
1	Yes	110 (37.2%)	186 (62.8%)	0.566	1.087	0.818–1.446			
Cook area	Cooking area								
0	Inside house	276 (33.5%)	547 (66.5%)						
1	Outside house	67 (51.1%)	64 (48.9%)	0.000	2.075	1.430–3.009			
Deworm	Deworming status								
0	Deworm	247 (33.7%)	487 (66.3%)						
1	Not deworm	96 (43.6%)	124 (56.4%)	0.007	1.526	1.123–2.076	0.006	1.676	1.161–2.424
Cow	Any cow								
0	No	296 (39.3%)	457 (60.7%)						
1	Yes	47 (23.4%)	154 (76.6%)	0.000	0.471	0.329–0.674	0.011	0.583	0.381–0.880
Smoking	Anyone smoke?								
0	Smoke	10 (16.4%)	51 (83.6%)						
1	Non-smoke	333 (37.3%)	560 (62.7%)	0.002	3.033	1.519–6.054	0.008	2.830	1.352–6.419
Cat	Do you have a cat?								
0	No	257 (38.6%)	408 (61.4%)						
1 (CatInside)	Lives inside	53 (25.4%)	156 (74.6%)	0.001	0.539	0.381–0.764			
2 (CatOutside)	Kept outside	33 (41.3%)	47 (58.7%)	0.652	1.115	0.695–1.786			
Dog	Do you have a dog								
0	No	228 (40.1%)	341 (59.9%)						
1 (DogInside)	Lives inside	0 (0%)	4 (100%)	0.998	0.000	0.000- inf			
2 (DogOutside)	Kept outside	115 (30.2%)	266 (69.8%)	0.002	0.647	0.491–0.852			
Elec	Electricity?								
0	Everyday	273 (55.2%)	222 (44.8%)						
1 (ElecSometimes)	Sometimes	11 (12.8%)	75 (87.2%)	0.000	0.124	0.064–0.239	0.007	0.363	0.167–0.734
2 (ElecNever)	Never	59 (15.8%)	314 (84.2%)	0.000	0.154	0.111–0.214	0.000	0.427	0.283–0.641
Floor	Type of floor								
0	Cement	150 (36.8%)	258 (63.2%)						
1 (WoodFloor)	Wood	4 (20%)	16 (80%)	0.202	0.434	0.121–1.563			
2 (MudFloor)	Mud	186 (35.9%)	332 (64.1%)	0.846	0.974	0.744–1.274			
9 (OtherFloor)	Others	3 (37.5%)	5(62.5%)	0.955	1.042	0.246–4.421			
Waste	Where do dispose waste								
0	Garbage bin	80 (44.2%)	101 (55.8%)						
1 (WastePit)	Pit	56 (36.6%)	97 (63.4%)	0.523	0.868	0.563–1.339			
2 (WasteField)	Open field	26 (12.6%)	181 (87.4%)	0.000	0.207	0.124–0.345	0.016	0.530	0.311–0.878
3 (WasteBurn)	Burning	181 (43.8%)	232 (56.2%)	0.405	1.156	0.822–1.626			
Years (Age)									
0	0–5	43 (47.3%)	48 (52.7%)						
1 (6–10Years)	6 to 10	167 (39.3%)	258 (60.7%)	0.176	0.735	0.471–1.148			
2 (11–15Years)	11 to 15	133 (30.4%)	305 (69.6%)	0.002	0.492	0.314–0.772			
FamSize	Family Size								
0	0–3	55 (34.6%)	104 (65.4%)						
1 (4-5FamSize)	4 or 5	165 (35.6%)	299 (64.4%)	0.616	1.100	0.758–1.595			
2 (> FamSize)	> 5	123 (37.2%)	208 (62.8%)	0.446	1.164	0.788–1.718	0.121	1.303	0.933–1.822
Toilet									
0	Flush toilet	10 (25%)	30 (75%)						
1 (ToiletPit)	Pit	325 (38.7%)	514 (61.3%)	0.109	1.738	0.885–3.415	0.004	2.340	1.337–4.244
2 (ToiletField)	Open field	8 (10.7%)	67 (89.3%)	0.027	0.328	0.122–0.881			
Water									
0	Piped	327 (38.3%)	526 (61.7%)						
1 (WaterWell)	Well	12 (15.4%)	66 (84.6%)	0.000	0.292	0.156–0.549			
2 (WaterNatural)	River/rain water	4 (17.4%)	19 (82.6%)	0.051	0.339	0.114–1.004			
HPYLORI		343 (36%)	611 (64%)						

### Classification model performance

**Fig. 2:**
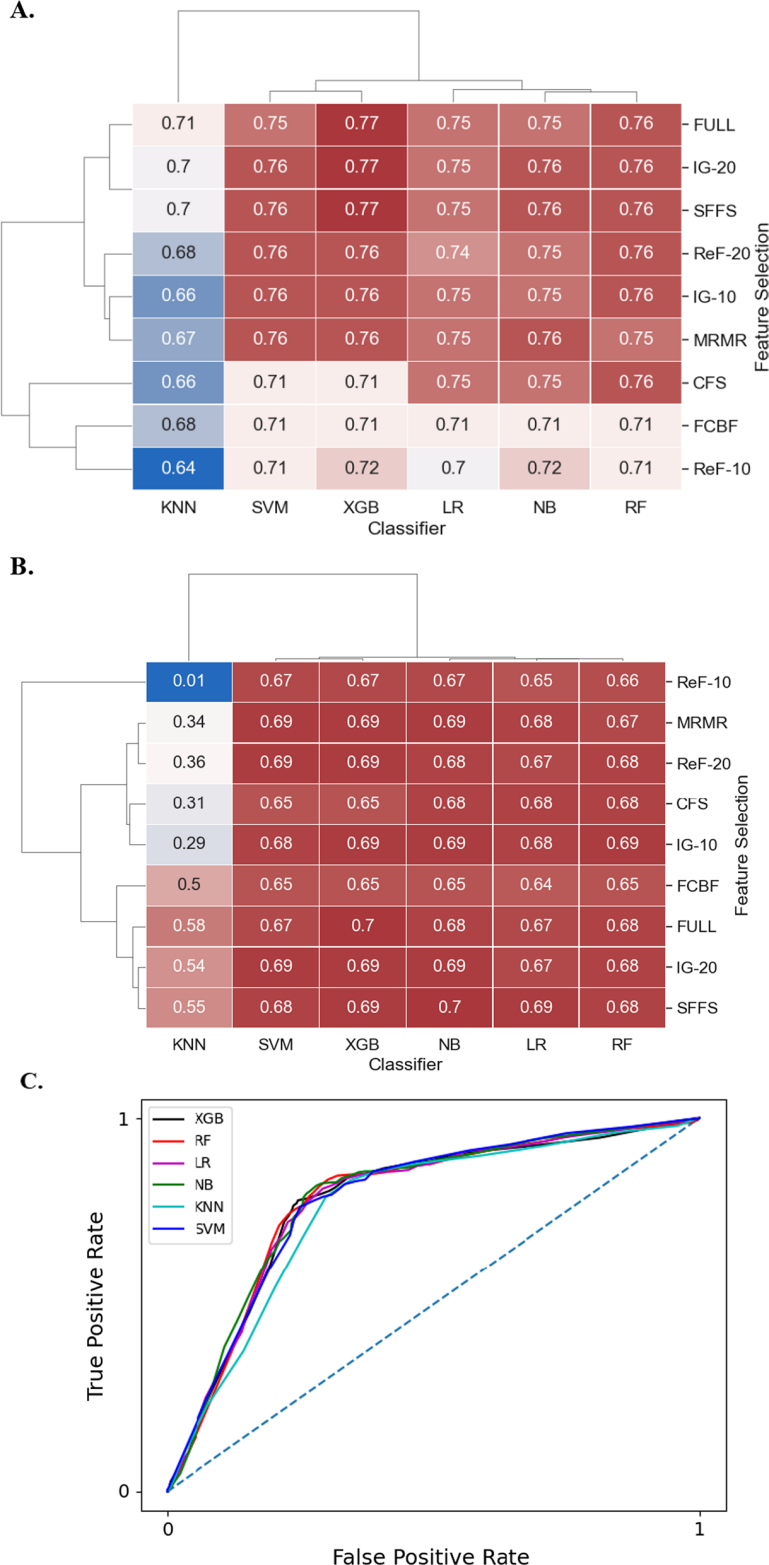
Average *H. Pylori* prevalence prediction accuracy and F1- scores of machine learning classifiers using various feature selection methods. Maroon and blue colors represent high and low accuracy (**A**), and F1 score (**B**), respectively. The numbers within each cell indicate the accuracy/F1-score of each classifier-feature selection method pair. KNN indicates K-Nearest Neighbors: SVM, Support Vector Machines; XGB, XGBoost; LR, Logistic Regression; NB, Naive Bayes; and RF, Random Forests. FULL indicates all risk factors are used. IG indicates Information Gain: ReF, ReliefF; MRMR, Minimum Redundancy Maximum Relevance; CFS, Correlation-based Feature Selection; FCBF, Fast Correlation Based Filter; and SFFS, Sequential Floating Forward Selection. The numbers -10 and -20 indicate the number of risk factors selected for the ranking-based feature selection methods. **C** The Receiver Operating Characteristic (ROC) curves of six classifiers (using their best hyperparameter combination) were obtained when they were used to predict *H. pylori* infection using a subset of risk factors selected through IG-20 feature selection method. The area under the ROC curve (AUROC) for KNN was 0.76, 0.79 for NB, and 0.78 for the other classifiers. The X-axis represents the False Positive Rate (1-Specificity) whereas the Y-axis represents the True Positive Rate (Sensitivity)

Among all classifiers, the XGB classifier had the highest accuracy score of 77% (Fig. 2A), up 13% from the baseline accuracy, guessing the most frequent class (*H. Pylori* negative.) SVM, NB, and RF showed comparable accuracy scores with XGB. However, KNN showed the worst performance across all classifiers methods. The logistic regression model, although comparable, showed slightly lower accuracy than all classifiers except KNN. The highest F1 score of 70% was reached by both NB and XGB classifiers, a 16% improvement from the baseline F1 score (Fig. 2B). The KNN classifier, again, had a relatively worse performance than other methods. All classifiers except KNN achieved similar F1 scores, regardless of the feature selection method used. Boosting and bagging methods did not significantly improve the accuracy or F1 score for any classifier (Additional file 1: Figs. S1 and S2). Figure 2C shows the model prediction performance using the area under the receiver operating characteristics curve (AUROC). XBG, NB, RF, SVM, and LR achieve 0.78–0.79 AUROC, slightly better than KNN's AUROC of 0.76. The results from both the nested cross validation (Additional file 1: Fig. S6) and on the synthetically up-sampled dataset (Additional file 1: Fig. S7) show similar accuracy levels and F1 scores. We have also provided the confusion matrix for the most accurate classifier-feature selection method combination in the supplement (Additional file 1: Table S1).

### *H. pylori* risk factors

**Fig. 3:**
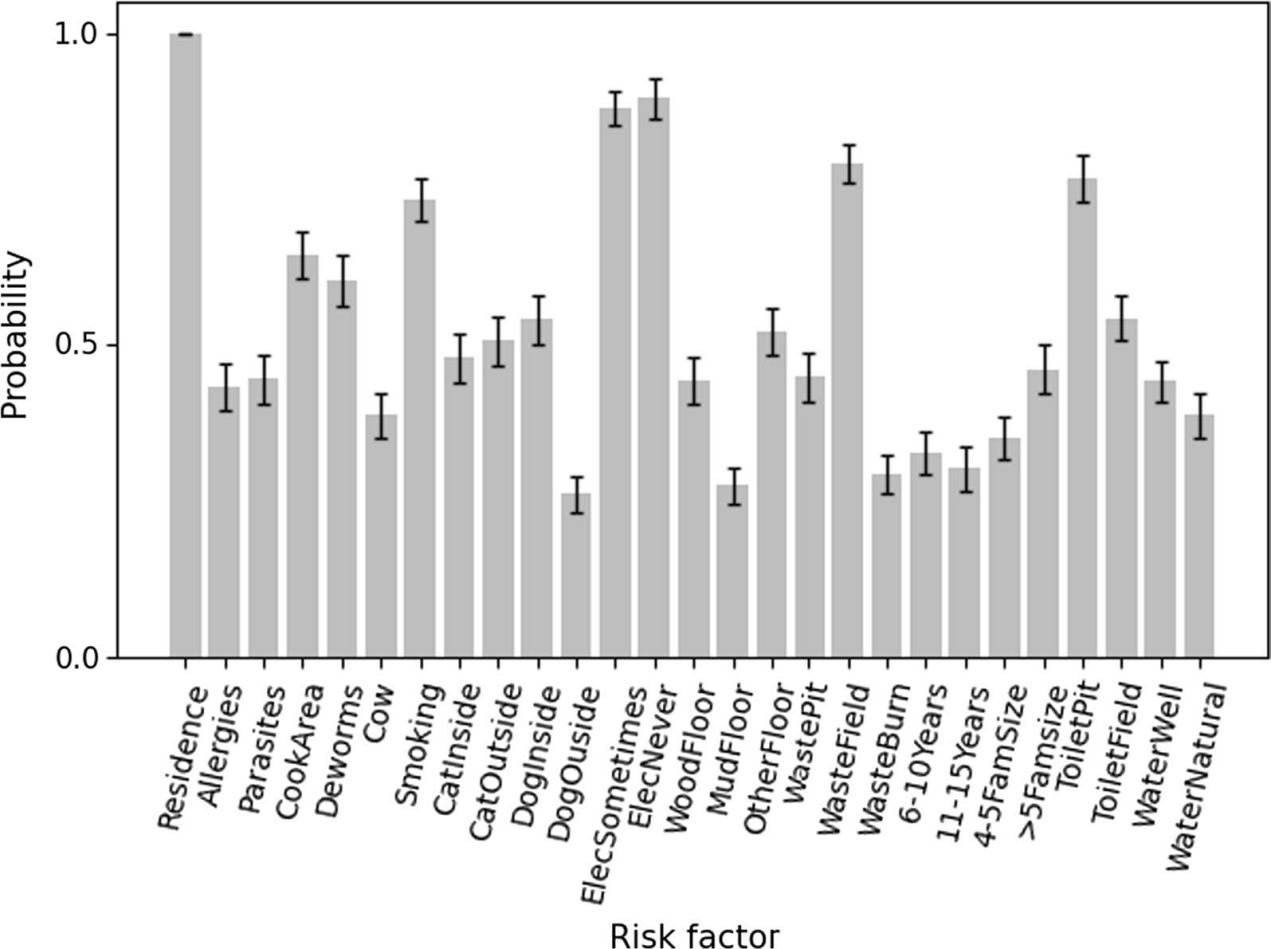
The relative importance of * H.pylori* risk factors based on all feature selection methods. X-axis indicates the *H. Pylori* risk factors, summarized in Table 1. Y-axis indicates the average probability of being selected across all feature selection methods. The error bars indicate one standard errors across all cross-validation folds

We derived the importance of *H. pylori* risk factors using feature selection methods in addition to univariate and multivariate logistic regression. We reported how frequently each feature (i.e., *H. pylori* risk factors) appeared in the fifty runs (five ten-fold cross-validations) of each feature selection method. The robustness of features selected across all feature selection methods is presented in Fig. 3 and Table 3. Additional file 1: Figs. S3, S4, and S5 show the robustness of each feature chosen for ranking, SFFS and subset-based feature selection methods, respectively.

**Table 3:** The frequency of *H. pylori risk* factors being chosen for all feature selection methods

Feature	All*	Multivariate LR
**Residence**	**100%(± 0%)**	X
Allergies	43.16%(± 3.82%)	
Parasites	44.42% ± (4.09%)	
*Cooking area*	*64.32%* ± *(3.74%)*	
*Dewormed status*	*60.42%(*± *4.11%)*	X
Cow	38.63%(± 3.58%)	X
*Smoking*	*73.26%(*± *3.5%)*	X
Cat: lives inside	47.89%(± 3.86%)	
***Cat: kept outside***	***50.63%(*± *3.91%)***	
***Dog: lives inside***	***54%(*± *3.84%)***	
Dog: kept outside	26%(± 2.78%)	
**Electricity use: sometimes**	**88%(± 2.71%)**	X
**Electricity use: never**	**89.47%(± 3.17%)**	X
Floor in home: wood	44.32%(± 3.78%)	
Floor in home: mud	27.37%(± 2.82%)	
***Floor in Home: Other***	***52%(*± *3.76%)***	
Waste disposal: pit	44.84%(± 3.88%)	
**Waste disposal: open field**	**79.05%(± 3.2%)**	X
Waste disposal: burn	29.26%(± 2.98%)	
Age: 6–10 years	32.63%(± 3.35%)	
Age: 11–15 years	30.11%(± 3.54%)	
Family size: 4–5	35.16%(± 3.47%)	
Family size: > 5	46%(± 3.94%)	
**Toilet: pit**	**76.74%(± 3.73%)**	X
***Toilet: open field***	***54.21%(*± *3.57%)***	
Water source: well	44.11%(± 3.38%)	
Water source: river or rain water	38.63%(± 3.45%)	

*Results from ranking-based, subset-based, and SFFS feature selection methods are combined. The features are indicated in the first column. The second column shows the average (± 1 standard error) frequency of being picked across all feature selection methods and cross-validation folds. The third column shows the features that the multivariate logistic regression approach determined to be significant. Bold, *italic*, and ***bold italic*** highlighted numbers show features that occur more frequently than **75 percent**, *60–75 percent*, and ***50–60 percent***, respectively

Among all features, place of residence (feature 1; with urban residence increasing risk) was found to be the most common risk factor of *H. pylori* infection, regardless of the feature selection method choice (Fig. 3). Over 75% of all feature selection runs also included four additional risk factors: sometimes or never using electricity (features 12–13, with always using electricity increasing risk), disposing of waste in an open field (feature 18), and using a pit for a toilet (feature 24). Thus, using a 75% robustness level, machine learning approaches identified five of the eight risk factors identified by traditional multivariate logistic regression (Table 3 and Additional file 1: Table S1). They do, however, identify three additional risk factors using a robustness level of 60%: presence of a smoker in the household (feature 7), place of cooking (feature 4; with cooking outside increasing risk), and mass deworming status (feature 5). Thus, feature selection methods identified seven of the eight risk factors identified by multivariate logistic regression. In multivariate logistic regression, the place of cooking (feature 4) is not found to be significant, and having a cow (feature 6) is not selected in more than 60% of feature selection runs. Finally, by employing a 50% robustness cutoff, the machine learning algorithms detect four additional features missed by multivariate logistic regression. The remaining fifteen features were not considered robust (i.e., were chosen by less than 50% of feature selection runs), including: keeping the family dog outside (feature 11), having mud floors (feature 15), disposing of waste by burning (feature 19), being 6–10 years old (feature 20), or being 11–15 years old (feature 21).

### Comparison of feature selection methods

**Fig. 4:**
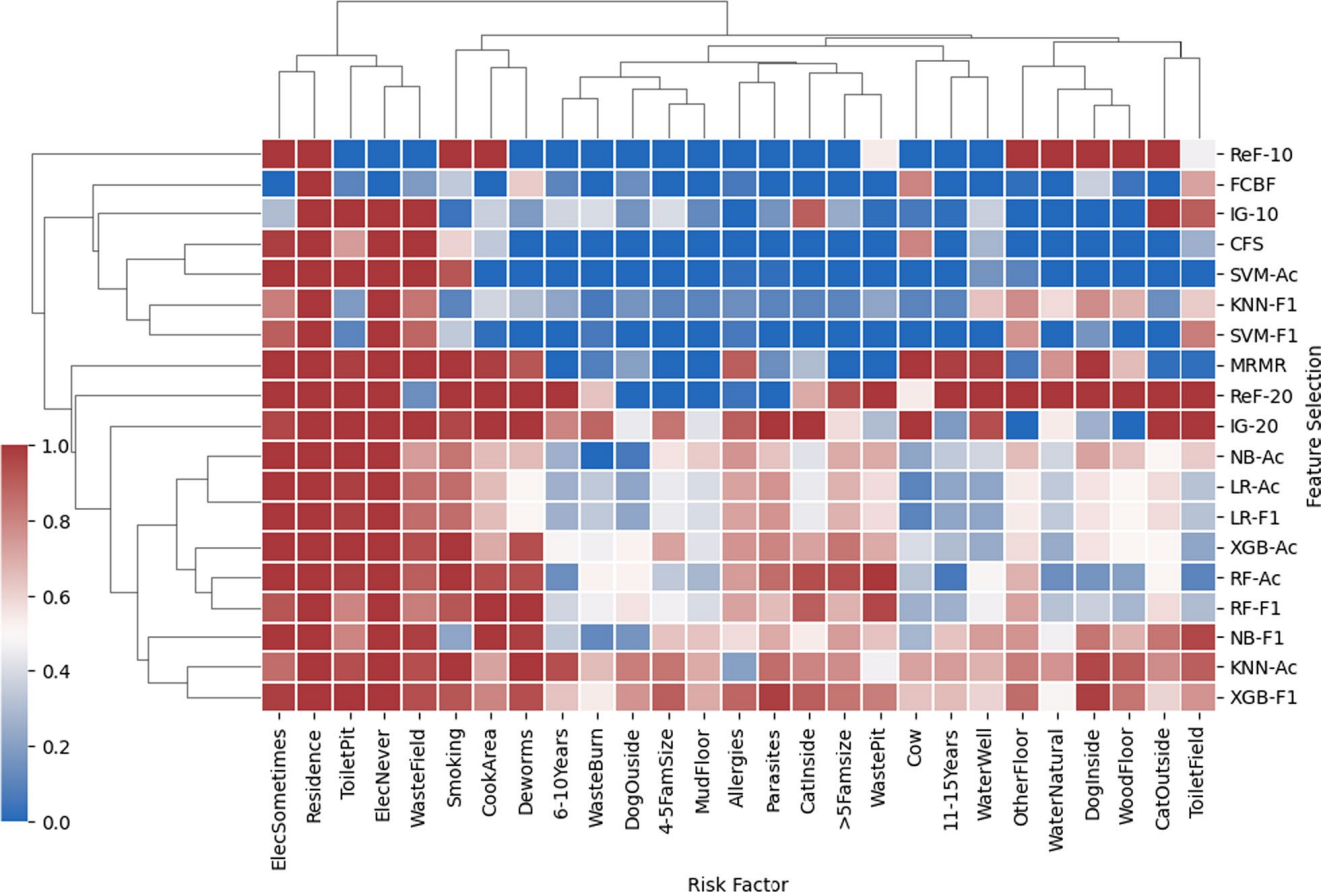
Two-dimensional hierarchical clustering heatmap of *H. pylori* risk factors and feature selection methods. Maroon and blue colors indicate more and less frequently selected features in five tenfold cross-validation runs, respectively. X-axis shows the *H*. *pylori* risk factors, summarized in Table 1. Y-axis indicates all feature selection methods. The risk factors found more frequently by feature selection methods appear on the heatmap's left columns. The feature selection methods that select the greatest number of risk factors appear on the heatmap's bottom rows. The risk factors grouped together suggest that they have been chosen similarly under varying feature selection methods. The feature selection methods grouped together indicate that these methods choose a similar set of risk factors

We discovered a clear distinction between selected and non-selected risk factors for all ranking-based (IG and ReF) and subset-based (CFS, FCBF, MRMR, and SFFS) methods (Fig. 4). Compared to other feature selection methods, the SFFS method exhibits higher variation between feature selection runs (Fig. 4, Additional file 1: Fig. S4). Among the SFFS results, KNN-accuracy and XGB-F1 performed differently than other SFFS results in terms of feature selection. These two methods selected more risk factors than the other SFFS runs. Using either accuracy or F1 score for the same classifier in SFFS runs results in classifier specific differences for the selected risk factors. For example, while the selected risk factors for RF-accuracy and RF-F1 are very similar, the risk factors selected for KNN-accuracy and KNN-F1 are different. We also observe that the selected risk factors vary significantly depending on the ranking- or subset-based method used. For example, comparing ReF-10 and IG-10, we see that having a dog living inside (feature 10), having a wood floor (feature 14), having a floor made of other materials (feature 16), and having rain or river water as a water source (feature 27) were always selected by ReF-10 but were never selected by IG-10.

## Discussion

We have employed a wide range of machine learning tools to assess the *H. pylori* risk factors in our data from a resource-limited setting. Several previous studies have demonstrated that in predictive tasks, machine learning classifiers do not outperform logistic regression[39–41]. Similarly, we demonstrated that, with the exception K-Nearest Neighbors, all classifiers showed comparable prediction performance. However, we would like to point out that the XGBoost method performed slightly better than other classifiers in predicting *H. pylori* prevalence, a 13% improvement over baseline accuracy. This finding is consistent with a previous study that found that XGB was more accurate than other machine learning classifiers in identifying HIV status using socio-behavioral-driven data [42].

Our finding that place of residence (feature 1) was the best predictor of *H. pylori* risk by all feature selection methods likely reflects the influence of socioeconomic status. Together with hygiene- and sanitation-related metrics like cooking area (feature 4) and having a pit toilet (feature 24), these findings corroborate previous epidemiological findings that suggest socioeconomic factors, hygiene, and sanitation are important predictors of *H. pylori* infections [43, 44].

Among the factors that increase the risk of having *H. pylori* infection, not having any smokers in the house seemed counter-intuitive, as having smokers in the house is often associated with disease susceptibility. However, in a setting like Ziway, Ethiopia, having smokers in the house might indicate that the family is in a good financial position and, therefore, better living conditions. Historically, smoking tends to be adopted by the socioeconomically advantaged classes before moving onto the disadvantaged groups and a positive association between smoking and socioeconomic status has been found in study sub-Saharan Africa [45]. Similarly, very low daily electricity consumption and living in a rural area appear to decrease the risk of *H. pylori* infection. However, we know that low electric consumption and rural residence usually do not indicate good socioeconomic standing. For example, a study has shown that one of the most significant determinants of energy consumption is household expenditure levels [44]. As such, we might need closer examination into the population we study. It is possible that most of the samples in our dataset from urban areas may be living in cramped, crowded slums, which exacerbate the likelihood of catching diseases. If that is the case, having access to and using electricity might merely be a good predictor of their social-economic status.

In this study, the traditional stepwise multivariate logistic regression analysis identified eight risk factors for *H. pylori* infection. In contrast, the machine learning algorithms identified five out of eight significant risk factors using a 75% robustness cutoff. However, by adjusting the robustness cut off value, machine learning algorithms capture nearly all the eight risk factors identified by the multivariate logistic regression, as well as several risk factors that were missed by the multivariate logistic regression but reported in other studies [46, 47]. Some of these features have been studied before: for example, contact with pet cats were found to be a statistically significant risk factor associated with *H.* pylori in children in Argentina [46]. Moreover, if a household keeps a cat outside then it might be indicative of the availability of space inside their house. Previous studies have shown that when pet ownership is positively correlated with socioeconomic status, it is also inversely correlated with prevalence of *H. pylori*[47]. Similarly, open field defecation has also been shown to be a strong indicator of *H. pylori* status, particularly when the point of waste disposal is located near homes [44]. Our results agree with previous study argued the ability of advanced machine learning approaches identifying unexpected predictor variables and possible connections that may be overlooked with a predefined hypothesis [48]. Furthermore, in population-based epidemiology studies where diverse socio-demographic variables are collected, an advanced ML approach allows for evaluation of far more variables than would be present in traditional modelling approaches. Additionally, our study showed that the relative importance of risk factors depended on the feature selection method used to select features.

The majority of the risk factors identified through feature selection also show up as significant in the traditional univariate and multivariate logistic regression analysis as well: with a mix of odd ratios greater than and less than 1. This suggests that the risk factors chosen by machine learning consist of a combination of additive and mitigative factors. We also see that factors that were insignificant in both the univariate and multivariate cases do not show up as robustly in the selected features through machine learning approaches. Since we have repeated our analysis with multiple feature selection methods, we have more confidence in the results since employing a variety of methods will likely reduce biases introduced by each technique. Moreover, the results from the nested cross validation suggests that our model is not overfit, and generalizable. Thus, our model can be used in similar epidemiological studies. Though, our machine learning methods should be retrained for different study settings.

Ultimately, by utilizing machine learning, we see that we might uncover patterns that may not be entirely apparent or add more reliability to the established methods used in epidemiology. Previous studies in other infectious diseases showed that using ML-based models identifies patients at risk of severe illness and Mortality from COVID-19 [49, 50], predict trend of tuberculosis incidence[51] and support of diagnosis and choice of appropriate antimicrobial treatment[52]. The authors suggested that ML models could contribute to public health workers or policymakers who need to identify high-risk populations and develop a prioritized treatment strategy accordingly. Similarly, the validated ML model in the study could be used in future databases to improve *H. pylori* risk prediction and targeted prevention efforts in the resource limited settings.

## Limitations

We might have participation bias and reporting bias in our data collection process. Some missing data values were imputed using an iterative imputer, which may introduce some noise into the data. We have also used an imputer when we repeated our study on an up-sampled dataset. While our classifiers can beat the baseline accuracy in predictive capacity, it is limited by the amount of data we have collected. As is the case with many machine learning techniques, we could elucidate much more from our analysis if we had more data points from a broader range of sources. We were also restricted by the amount of processing power needed to run some of these algorithms. Given more computational resources, we might be able to exceed our current predictive limit. Finally, while machine learning methods can assist us in determining the importance of specific risk factors, they do not always show the directionality of the association.

## Conclusion

Our machine learning-based approach achieved substantial gains in predictive accuracy over the baseline and has shed light on various expected and unexpected risk factors that influence *H. pylori* infection.

## Supplementary Information

**Additional file 1: Figure S1.** Average *H. pylori* prediction accuracy and F1 scores for each classifier and feature selection pair with boosting. **Figure S2.** Average *H. pylori* prediction accuracy and F1 score for each classifier and feature selection pair with bagging. **Figure S3.** The probability of each feature being selected averaged across all ranking-based methods with standard error. **Figure S4.** *H. pylori* risk factors' relative importance based on classifier-accuracy and F1-score based SFFS feature selection. **Figure S5.** The probability of each feature being selected averaged across all subset-based methods with standard error. **Figure S6.** Average *H. pylori* prevalence prediction accuracy and F1- scores of machine learning classifiers using various feature selection methods and nested cross validation. **Table S1.** Confusion matrix for the best performing model in terms of predictive accuracy in the nested cross validation. **Figure S7. **Average *H. pylori* prevalence prediction accuracy and F1-scores of machine learning classifiers using various feature selection methods on dataset upscaled with SMOTE.

## Data Availability

The datasets generated and/or analyzed during the current study are not publicly available because the local ethics review committee require oversight of use of research data but are available from the corresponding author on reasonable request.

## References

[1] Miernyk KM, Bulkow LR, Gold BD, Bruce MG, Hurlburt DH, Griffin PM (2018). Prevalence of Helicobacter pylori among Alaskans: Factors associated with infection and comparison of urea breath test and anti-Helicobacter pylori IgG antibodies. Helicobacter.

[2] Eshraghian A (2014). Epidemiology of Helicobacter pylori infection among the healthy population in Iran and countries of the Eastern Mediterranean Region: A systematic review of prevalence and risk factors. World J Gastroenterol.

[3] Łaszewicz W, Iwańczak F, Iwańczak B, Annabhani A, Bała G, Bąk-Romaniszyn L (2014). Seroprevalence of Helicobacter pylori infection in Polish children and adults depending on socioeconomic status and living conditions. Adv Med Sci.

[4] Mathewos B, Moges B, Dagnew M (2013). Seroprevalence and trend of Helicobacter pylori infection in Gondar University Hospital among dyspeptic patients, Gondar, North West Ethiopia. BMC Res Notes.

[5] Smith S, Jolaiya T, Fowora M, Palamides P, Ngoka F, Bamidele M (2018). Clinical and Socio- Demographic Risk Factors for Acquisition of Helicobacter pylori Infection in Nigeria. Asian Pac J Cancer Prev.

[6] Ueda M, Kikuchi S, Kasugai T, Shunichi T, Miyake C (2003). Helicobacter pylori risk associated with childhood home environment. Cancer Sci.

[7] Porras C, Nodora J, Sexton R, Ferreccio C, Jimenez S, Dominguez RL (2013). Epidemiology of Helicobacter pylori infection in six Latin American countries (SWOG Trial S0701). Cancer causes & control : CCC.

[8] Klein PD, Opekun AR, Smith EO, Klein PD, Graham DY, Graham DY (1991). Water source as risk factor for Helicobacter pylori infection in Peruvian children. The Lancet.

[9] Awuku YA, Simpong DL, Alhassan IK, Tuoyire DA, Afaa T, Adu P (2017). Prevalence of helicobacter pylori infection among children living in a rural setting in Sub-Saharan Africa. BMC Public Health.

[10] Ozbey G, Hanafiah A (2017). Epidemiology, diagnosis, and risk factors of helicobacter pylori infection in children. Euroasian J Hepatogastroenterol.

[11] Dore MP, Malaty HM, Graham DY, Fanciulli G, Delitala G, Realdi G (2002). Risk factors associated with *Helicobacter pylori* infection among children in a defined geographic area. Clin Infect Dis.

[12] Braga ABC, Fialho AMN, Rodrigues MN, Queiroz DMM, Rocha AMC, Braga LLBC (2007). *Helicobacter pylori* colonization among children up to 6 years: results of a community-based study from Northeastern Brazil. J Trop Pediatr.

[13] Owyang SY, Luther J, Kao JY (2011). *Helicobacter pylori*: beneficial for most?. Expert Rev Gastroenterol Hepatol.

[14] Cover TL, Blaser MJ (2009). Helicobacter pylori in health and disease. Gastroenterology.

[15] Schacher K, Spotts H, Correia C, Walelign S, Tesfaye M, Desta K (2020). Individual and household correlates of Helicobacter pylori infection among Young Ethiopian children in Ziway, Central Ethiopia. BMC Infect Dis.

[16] Dreiseitl S, Ohno-Machado L (2002). Logistic regression and artificial neural network classification models: a methodology review. J Biomed Inform.

[17] Deo RC (2015). Machine learning in medicine. Circulation.

[18] Baxendell K, Walelign S, Tesfaye M, Wordofa M, Abera D, Mesfin A (2019). Association between infection with Helicobacter pylori and platelet indices among school-aged children in central Ethiopia: a cross-sectional study. BMJ Open.

[19] Mohamed N, Muse A, Wordofa M, Abera D, Mesfin A, Wolde M (2019). Increased Prevalence of Cestode Infection Associated with History of Deworming among Primary School Children in Ethiopia. Am J Trop Med Hyg.

[20] Quinlan JR (1986). Induction of decision trees. Mach Learn.

[21] Robnik-Šikonja M, Kononenko I, editors. An adaptation of Relief for attribute estimation in regression. Machine Learning: Proceedings of the Fourteenth International Conference (ICML’97); 1997.

[22] Hall MA. Correlation-based feature selection of discrete and numeric class machine learning. 2000.

[23] Pudil P, Novovičová J, Kittler J (1994). Floating search methods in feature selection. Pattern Recogn Lett.

[24] Yu L, Liu H, editors. Feature selection for high-dimensional data: A fast correlation-based filter solution. Proceedings of the 20th international conference on machine learning (ICML-03); 2003.

[25] Ding C, Peng H (2005). Minimum redundancy feature selection from microarray gene expression data. J Bioinform Comput Biol.

[26] Chen T, Guestrin C, editors. Xgboost: A scalable tree boosting system. Proceedings of the 22nd ACM SIGKDD international conference on knowledge discovery and data mining; 2016.

[27] Breiman L (2001). Random Forests. Mach Learn.

[28] John GH, Langley P. Estimating continuous distributions in Bayesian classifiers. arXiv preprint arXiv:13024964. 2013.

[29] Aha DW, Kibler D, Albert MK (1991). Instance-based learning algorithms. Mach Learn.

[30] Vapnik V (2013). The nature of statistical learning theory.

[31] Tibshirani R (1996). Regression Shrinkage and Selection via the Lasso. J Roy Stat Soc: Ser B (Methodol).

[32] Freund Y, Schapire RE, editors. Experiments with a new boosting algorithm. ICML; 1996: Citeseer.

[33] Breiman L (1996). Bagging predictors. Mach Learn.

[34] Chawla NV, Bowyer KW, Hall LO, Kegelmeyer WP (2002). SMOTE: synthetic minority over-sampling technique. J Artif Intell Res.

[35] Bishop CM (2006). Pattern recognition and machine learning.

[36] Hastie T, Tibshirani R, Friedman J (2009). Random forests. The elements of statistical learning.

[37] van Buuren S, Groothuis-Oudshoorn K (2011). Mice: multivariate imputation by chained equations in R. JStat Softw.

[38] Rokach L, Maimon O (2005). Clustering methods. Data mining and knowledge discovery handbook.

[39] Christodoulou E, Ma J, Collins GS, Steyerberg EW, Verbakel JY, Van Calster B (2019). A systematic review shows no performance benefit of machine learning over logistic regression for clinical prediction models. J Clin Epidemiol.

[40] van der Ploeg T, Austin PC, Steyerberg EW (2014). Modern modelling techniques are data hungry: a simulation study for predicting dichotomous endpoints. BMC Med Res Methodol.

[41] Jiang Y, Zhang X, Ma R, Wang X, Liu J, Keerman M (2021). Cardiovascular disease prediction by machine learning algorithms based on cytokines in Kazakhs of China. Clin Epidemiol.

[42] Mutai CK, McSharry PE, Ngaruye I, Musabanganji E. Use of machine learning techniques to identify HIV predictors for screening in sub-Saharan Africa. BMC Med Res Methodol. 2021;21(1):159. 10.1186/s12874-021-01346-2. 10.1186/s12874-021-01346-2 PMC8325403 34332540

[43] Smith S, Jolaiya T, Fowora M, Palamides P, Ngoka F, Bamidele M (2018). Clinical and socio- demographic risk factors for acquisition of helicobacter pylori infection in Nigeria. APJCP.

[44] Nurgalieva ZZ, Malaty HM, Graham DY, Almuchambetova R, Machmudova A, Kapsultanova D (2002). Helicobacter pylori infection in Kazakhstan: effect of water source and household hygiene. Am J Trop Med Hyg.

[45] Strebel P, Kuhn L, Yach D (1989). Determinants of cigarette smoking in the black township population of Cape Town. J Epidemiol Community Health.

[46] Goldman C, Barrado A, Janjetic M, Balcarce N, Cueto Rua E, Oshiro M (2006). Factors associated with *H. pylori* epidemiology in symptomatic children in Buenos Aires, Argentina. World J Gastroenterol.

[47] Graham DY, Malaty HM, Evans DG, Evans DJ, Klein PD, Adam E (1991). Epidemiology of *Helicobacter pylori* in an asymptomatic population in the United States. Effect of age, race, and socioeconomic status. Gastroenterology.

[48] Parikh RB, Manz C, Chivers C, Regli SH, Braun J, Draugelis ME (2019). Machine learning approaches to predict 6-month mortality among patients with cancer. JAMA Netw Open.

[49] Liang W, Yao J, Chen A, Lv Q, Zanin M, Liu J (2020). Early triage of critically ill COVID-19 patients using deep learning. Nat Commun.

[50] Hu C, Liu Z, Jiang Y, Shi O, Zhang X, Xu K (2021). Early prediction of mortality risk among patients with severe COVID-19, using machine learning. Int J Epidemiol.

[51] Mohammed SH, Ahmed MM, Al-Mousawi AM, Azeez A (2018). Seasonal behavior and forecasting trends of tuberculosis incidence in Holy Kerbala, Iraq. Int J Mycobacteriol.

[52] Peiffer-Smadja N, Rawson TM, Ahmad R, Buchard A, Georgiou P, Lescure FX (2020). Machine learning for clinical decision support in infectious diseases: a narrative review of current applications. Clin Microbiol Infect.

