# Supplementary material for: Helicobacter pylori (H. pylori) risk factor analysis and prevalence prediction: a machine learning-based approach
Source: BMC Infect Dis. 2022 Jul 28;22:655. doi: 10.1186/s12879-022-07625-7 (PMC9330977; doi:10.1186/s12879-022-07625-7)
Supplement: Supplementary file 1 — Additional file 1: Figure S1. Average H. pylori prediction accuracy and F1 scores for each classifier and feature selection pair with boosting. Figure S2. Average H. pylori prediction accuracy and F1 score for each classifier and feature selection pair with bagging. Figure S3. The probability of each feature being selected averaged across all ranking-based methods with standard error. Figure S4. H. pylori risk factors' relative importance based on classifier-accuracy and F1-score based SFFS feature selection. Figure S5. The probability of each feature being selected averaged across all subset-based methods with standard error. Figure S6. Average H. pylori prevalence prediction accuracy and F1- scores of machine learning classifiers using various feature selection methods and nested cross validation. Table S1. Confusion matrix for the best performing model in terms of predictive accuracy in the nested cross validation. Figure S7. Average H. pylori prevalence prediction accuracy and F1-scores of machine learning classifiers using various feature selection methods on dataset upscaled with SMOTE. [file 12879_2022_7625_MOESM1_ESM.docx]

**Additional file 1 Figures**

**Additional file 1: Figure S1.**

**A.**

**
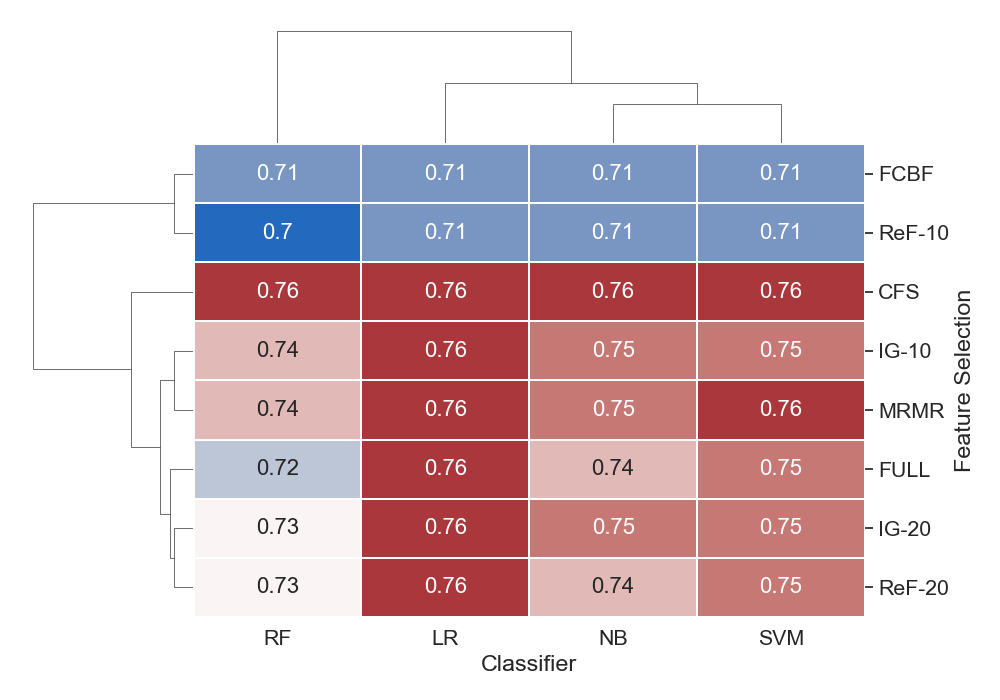
**

**B.**


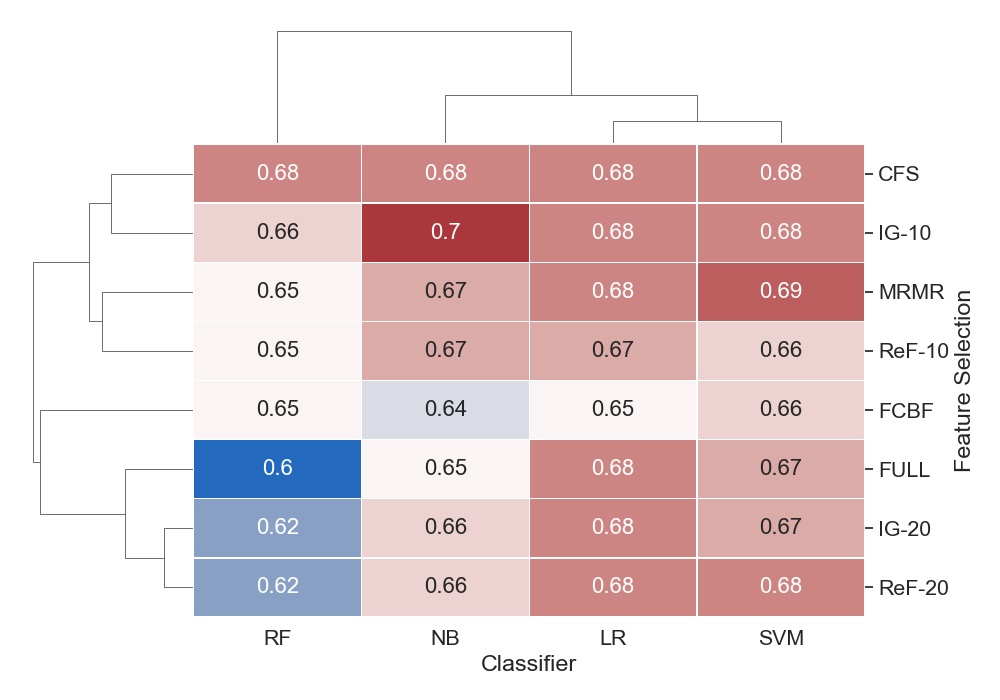


**Additional file 1: Figure S1. Average *H.pylori* prediction accuracy and F1 scores for each classifier and feature selection pair with boosting**. Maroon and blue colors represent high and low accuracy (A) and F1 score (B), respectively. The numbers within each cell indicate the accuracy/F1 score of each classifier-feature selection pair. SVM indicates Support Vector Machines: LR, Logistic Regression; NB, Naive Bayes; and RF, Random Forests. FULL indicates all risk factors are used. IG indicates Information Gain: ReF, ReliefF; MRMR, Minimum Redundancy Maximum Relevance; CFS, Correlation-based Feature Selection; and FCBF, Fast Correlation Based Filter. The numbers _10 and _20 indicate the number of risk factors selected for the ranking-based feature selection methods.

**Additional file 1: Figure S2.**

**A.**


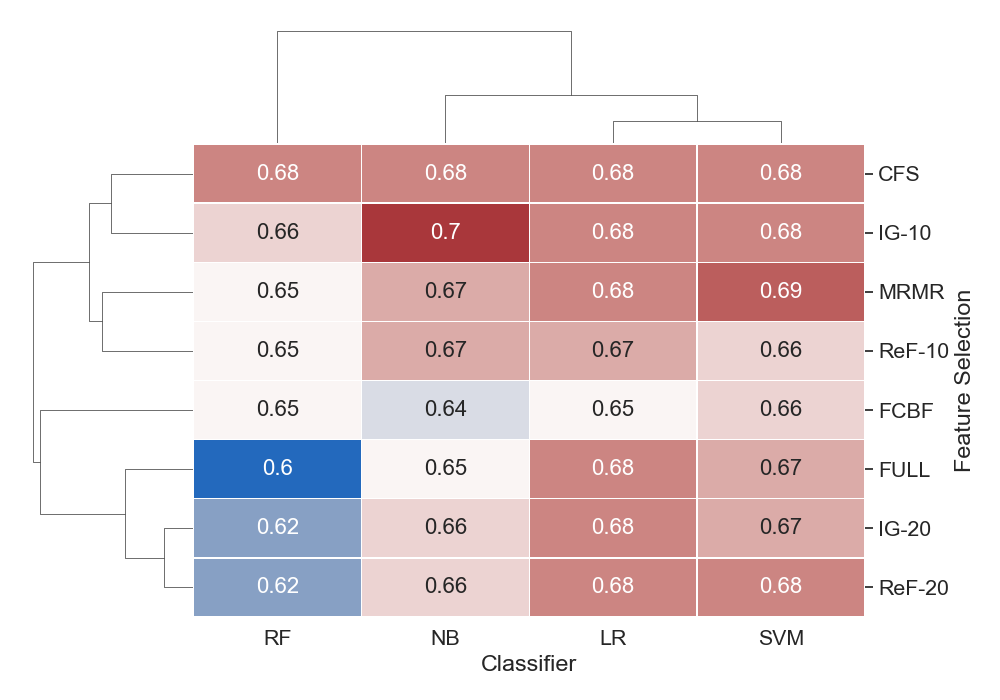


**B.**


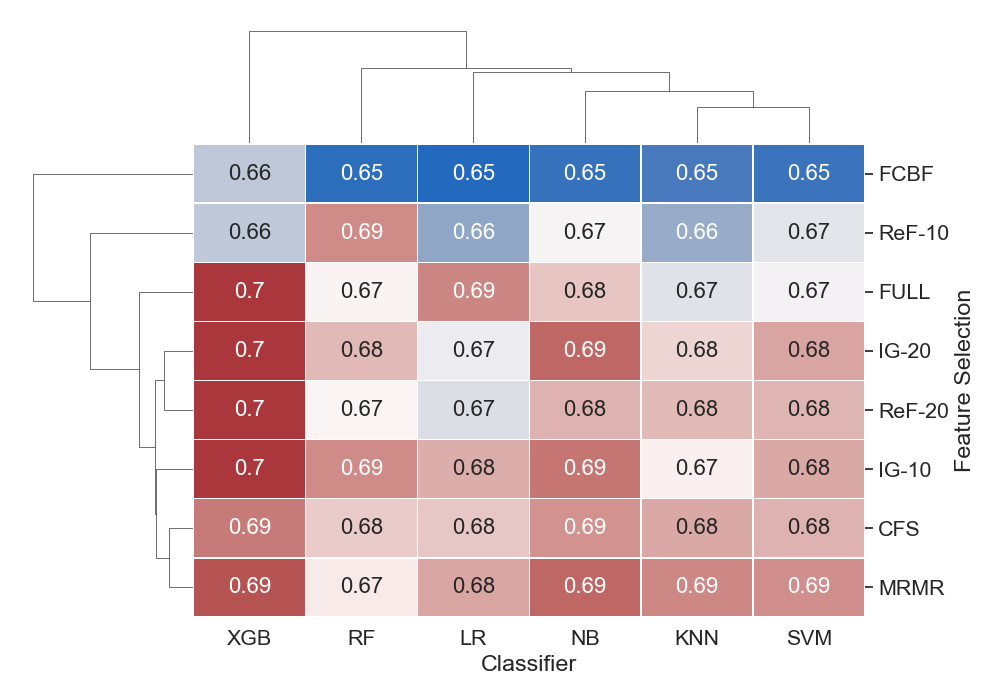


**Additional file 1: Figure S2. Average *H.pylori* prediction accuracy and F1 score for each classifier and feature selection pair with bagging.** Maroon and blue colors represent high and low accuracy (A) and F1 score (B), respectively. The numbers within each cell indicate the accuracy of each classifier-feature selection pair. SVM indicates Support Vector Machines: LR, Logistic Regression; NB, Naive Bayes; and RF, Random Forests. FULL indicates all risk factors are used. IG indicates Information Gain: ReF, ReliefF; MRMR, Minimum Redundancy Maximum Relevance; CFS, Correlation-based Feature Selection; and FCBF, Fast Correlation Based Filter. The numbers -10 and -20 indicate the number of risk factors selected for the ranking-based feature selection methods.

**Additional file 1: Figure S3.**

**
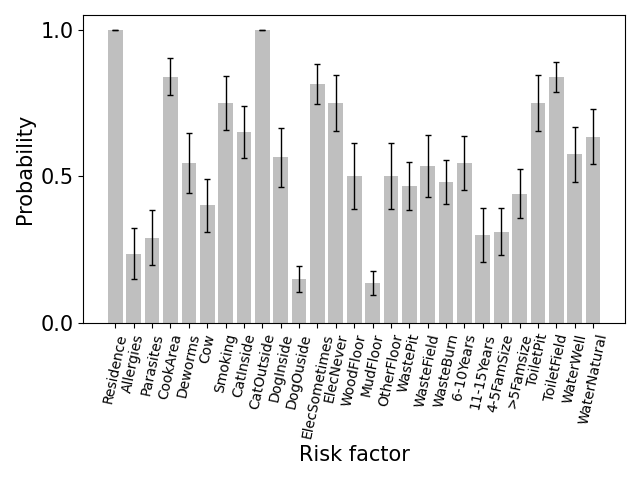
**

**Additional file 1: Figure S3. The probability of each feature being selected averaged across all ranking-based methods with standard error**. Ranking-based methods include IG (Information Gain) and ReF (ReliefF). X-axis indicates the features, and y-axis indicates the average probability of being selected across ranking-based methods. The error bars indicate one standard error across all cross-validation folds.

**Additional file 1: Figure S4.**

**A.**

**
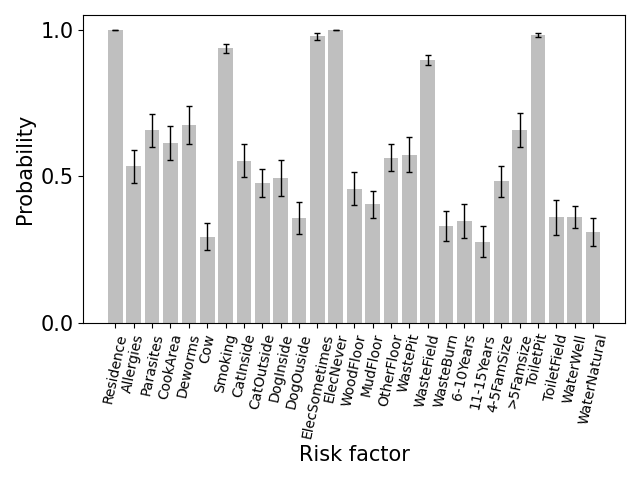
**

**B.**


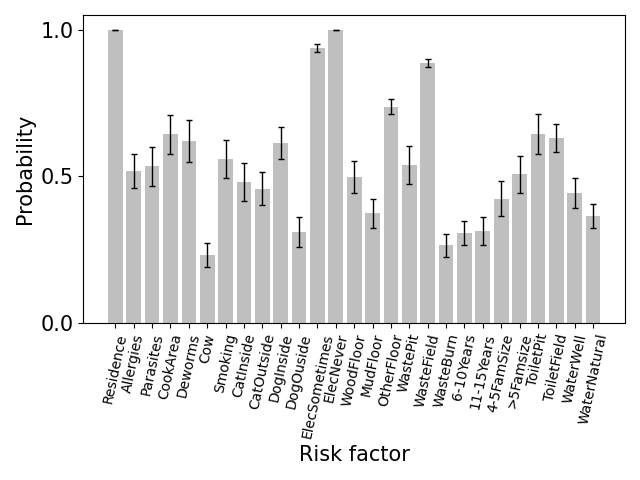


**Additional file 1: Figure S4. *H. Pylori* risk factors' relative importance based on classifier-accuracy (A) and F1-score (B) based SFFS feature selection.** Classifiers include K-Nearest Neighbors(KNN), Support Vector Machines(SVM), XGBoost(XGB), Naive Bayes(NB), Logistic Regression(LR), and Random Forests(RF). X-axis indicates the *H. Pylori* risk factors, summarized in Table 1. Y-axis indicates the average probability of being selected across all feature selection methods. The error bars indicate one standard error across all cross-validation folds.

**Additional file 1: Figure S5.**

**
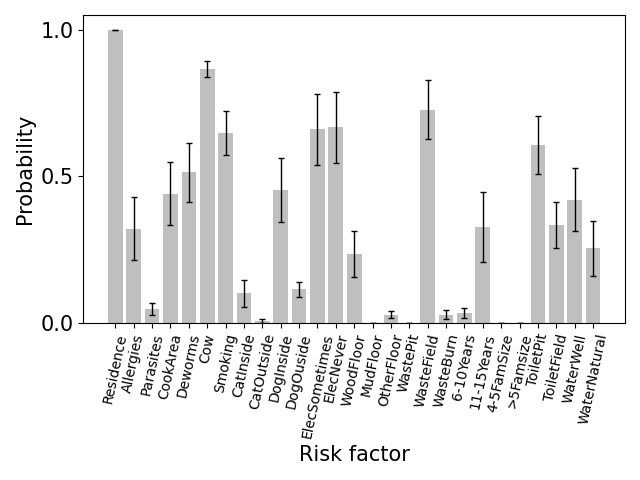
**

**Additional file 1: Figure S5. The probability of each feature being selected averaged across all subset-based methods with standard error**. Subset-based methods include CFS (Correlation-based Feature Selection), MRMR (Minimum Redundancy Maximum Relevance), and FCBF (Fast Correlation Based Filter), and y-axis indicates the average probability of being selected across subset-based methods. The error bars indicate one standard error across all cross-validation folds.

**Additional file 1: Figure S6.**

**A.**

**
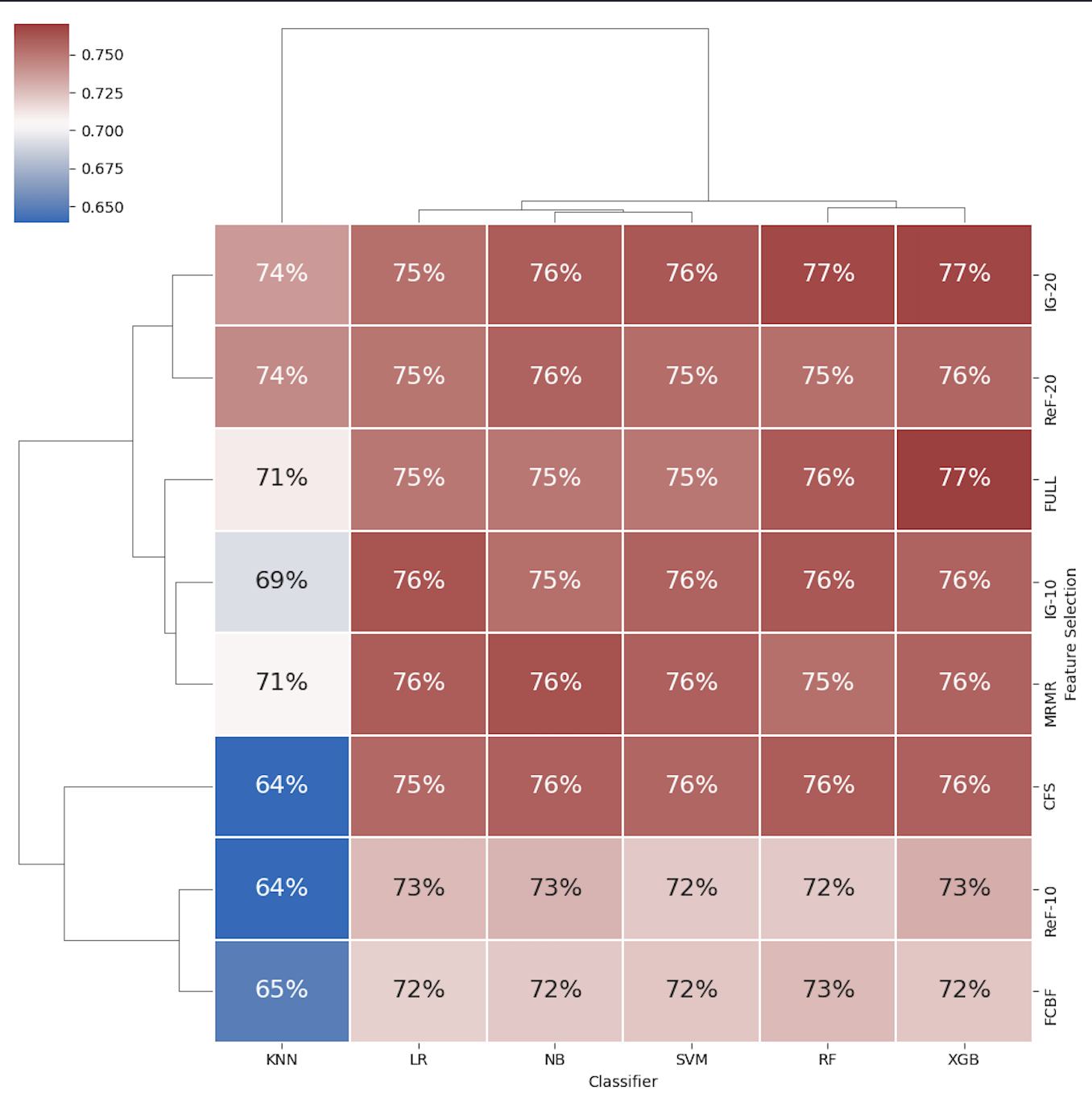
**

**B.**

**
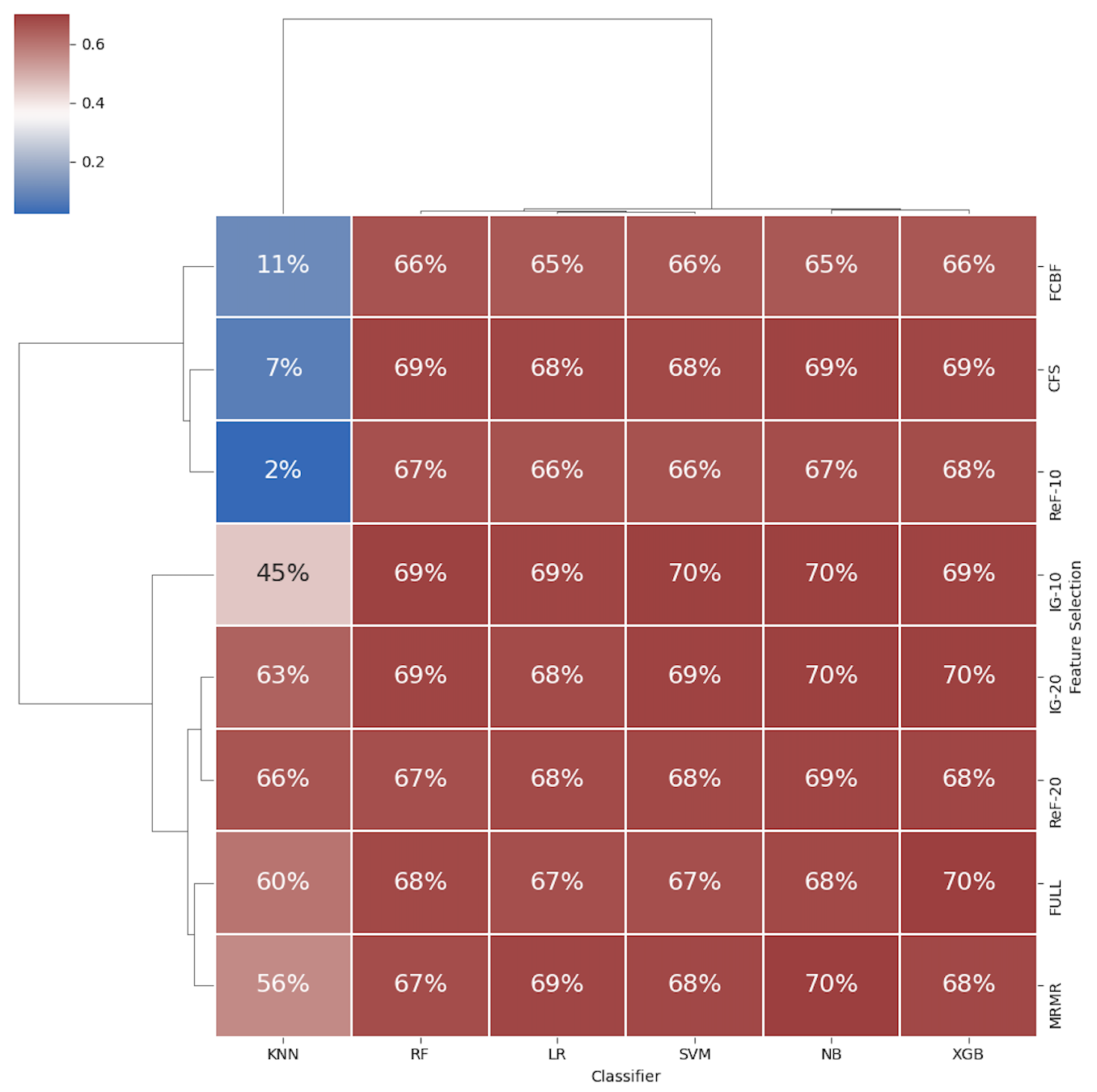
**

**Additional file 1: Figure S6. Average *H. Pylori* prevalence prediction accuracy and F1- scores of machine learning classifiers using various feature selection methods and nested cross validation.** Maroon and blue colors represent high and low accuracy (A), and F1 score (B), respectively. The numbers within each cell indicate the accuracy/F1-score of each classifier-feature selection method pair. KNN indicates K-Nearest Neighbors: SVM, Support Vector Machines; XGB, XGBoost; LR, Logistic Regression; NB, Naive Bayes; and RF, Random Forests. FULL indicates all risk factors are used. IG indicates Information Gain: ReF, ReliefF; MRMR, Minimum Redundancy Maximum Relevance; CFS, Correlation-based Feature Selection; FCBF, Fast Correlation Based Filter. The numbers -10 and -20 indicate the number of risk factors selected for the ranking-based feature selection methods.

**Additional file 1: Table S1.**

| **Classifier** | **Feature Selection** | **True Negative** | **False Positive** | **False Negative** | **True Positive** |
| --- | --- | --- | --- | --- | --- |
| K-Nearest Neighbors | ReF - 20 | 472 | 139 | 106 | 237 |
| Logistic Regression | IG - 10 | 483 | 128 | 99 | 244 |
| Naive Bayes | MRMR | 464 | 147 | 79 | 264 |
| Random Forests | IG - 20 | 488 | 123 | 100 | 243 |
| SVM | FCBF | 441 | 170 | 95 | 248 |
| XGBoost | IG - 20 | 477 | 134 | 88 | 255 |

**Additional file 1: Table S1. Confusion matrix for the best performing model in terms of predictive accuracy in the nested cross validation.** IG indicates Information Gain: ReF, ReliefF; MRMR, Minimum Redundancy Maximum Relevance; CFS, Correlation-based Feature Selection. The numbers -10 and -20 indicate the number of risk factors selected for the ranking-based feature selection methods.

**Additional file 1: Figure S7.**

**A.**

**
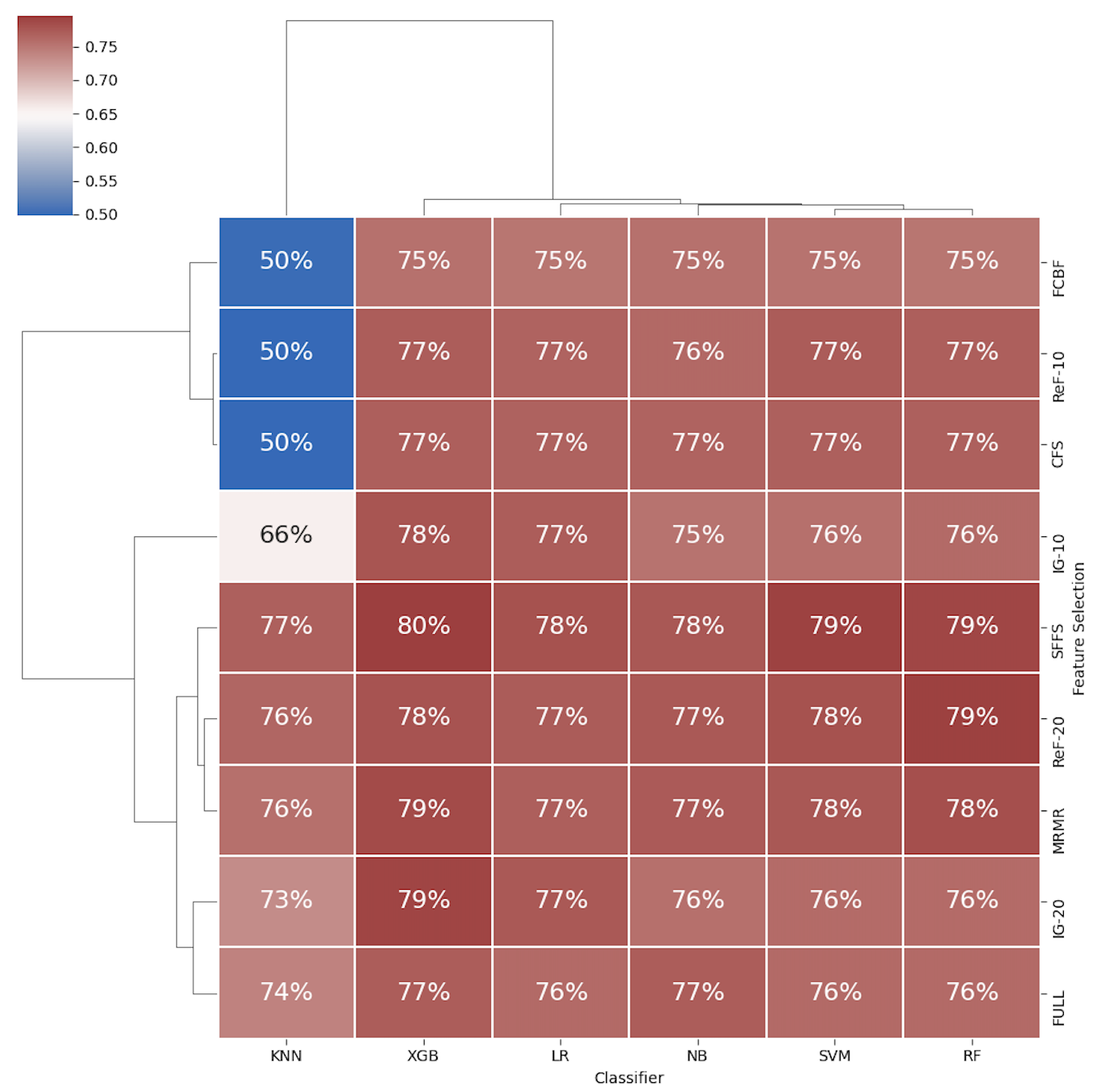
**

**B.**

**
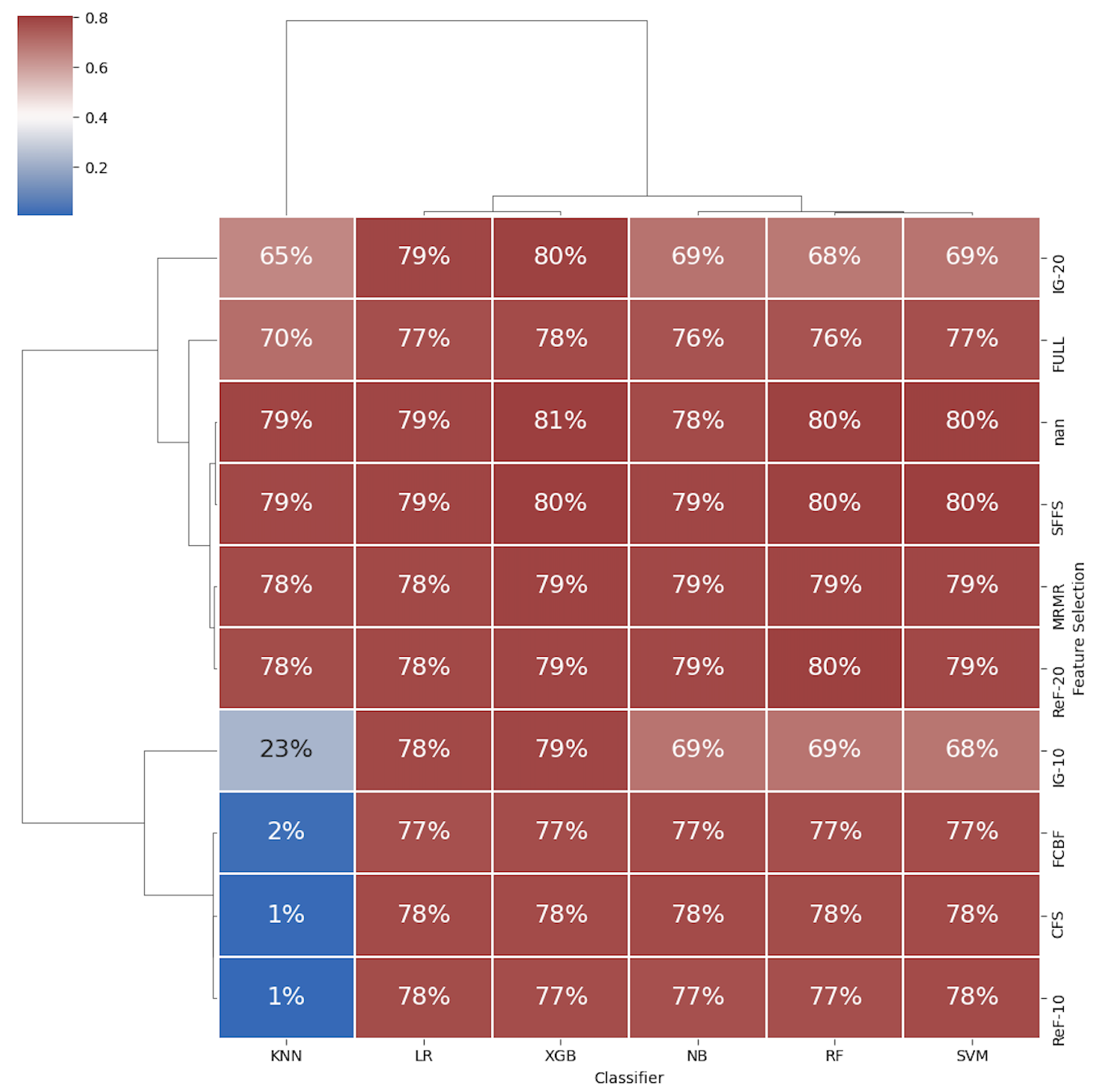
**

**Additional file 1: Figure S7. Average *H. Pylori* prevalence prediction accuracy and F1- scores of machine learning classifiers using various feature selection methods on dataset upscaled with SMOTE.** Maroon and blue colors represent high and low accuracy (A), and F1 score (B), respectively. The numbers within each cell indicate the accuracy/F1-score of each classifier-feature selection method pair. KNN indicates K-Nearest Neighbors: SVM, Support Vector Machines; XGB, XGBoost; LR, Logistic Regression; NB, Naive Bayes; and RF, Random Forests. FULL indicates all risk factors are used. SFFS indicates Sequential Forward Feature Selector; IG indicates Information Gain: ReF, ReliefF; MRMR, Minimum Redundancy Maximum Relevance; CFS, Correlation-based Feature Selection; FCBF, Fast Correlation Based Filter. The numbers -10 and -20 indicate the number of risk factors selected for the ranking-based feature selection methods.
